# The predictive value of revised diastolic dysfunction in outcomes of liver transplantation: A propensity score matching analysis

**DOI:** 10.3389/fsurg.2022.1072908

**Published:** 2023-01-06

**Authors:** Shenghua Bi, Yueping Jiang, Wenjun Zhao, Xiaoyan Niu, Xuechun Liu, Xue Jing

**Affiliations:** ^1^ Gastroenterology Department, The Affiliated Hospital of Qingdao University, Qingdao, China; ^2^ Department of Ultrasound, The Affiliated Hospital of Qingdao University, Qingdao, China

**Keywords:** decompensated cirrhosis, cirrhotic cardiomyopathy, liver transplantation, adverse events, mortality

## Abstract

**Background:**

Diastolic dysfunction (DD), one of the earliest signs of cirrhotic cardiomyopathy (CCM), is included in the revised 2019 CCM criteria. Nonetheless, relevant research regarding the effects of revised DD on post-liver transplantation (LT) outcomes remains limited.

**Methods:**

This retrospective study enrolled patients who underwent LT for decompensated cirrhosis, from January 2018 to March 2021. Patients were divided into DD and non-DD groups. Clinical data were collected. Patients were followed up with, for at least 1 year post-LT; cardiovascular adverse events (AEs) and survival status were recorded. Risk factors were identified using 1:2 propensity score matching (PSM), after adjusting for confounding factors. The caliper value was set to 0.02.

**Results:**

Of 231 patients, 153 were diagnosed with DD (male, 81.8%; mean age, 51.5 ± 9.5 years). Nineteen patients with DD died within 1 year, post-LT. After PSM, 97 and 60 patients were diagnosed with and without DD, respectively. Patients with DD had longer intensive care unit (ICU) stays, higher perioperative cardiovascular AEs, and higher mortality rates than those without DD. In a multivariate analysis, interventricular septum (IVS), left atrial volume index (LAVI), and potassium levels were independent prognostic factors of perioperative cardiovascular AEs, while a decreased early diastolic mitral annular tissue velocity (e’), increased neutrophil-to-lymphocyte ratio (NLR) and tumor markers were predictors of mortality within 1 year post-LT after PSM (*P* < 0.05).

**Conclusion:**

Cardiac DD may contribute to perioperative cardiovascular AEs and mortality post-LT. Clinicians should be aware of decompensated cirrhosis in patients with DD.

## Introduction

Cirrhotic cardiomyopathy (CCM) is defined as impaired contractility due to stress and/or diastolic dysfunction (DD) with electrophysiological abnormalities (1, 2), which is associated with a high incidence of complications and poor survival after liver transplantation (LT) (3, 4). The prevalence of CCM is approximately 33%–53% in patients on the transplant waiting list (5). Some studies have shown that early to late diastolic trans-mitral flow velocity (E/A), isovolumetric relaxation time (IVRT), and time-delay estimation (TDE) are prognostic markers of CCM (6).

DD, as one of the earliest signs of CCM, was entirely updated in the revised 2019 criteria (7) of the Cirrhotic Cardiomyopathy Consortium (CCC), including septal early diastolic mitral annular tissue velocity (e’), early diastolic trans-mitral flow to early diastolic mitral annular tissue velocity (E/e’), left atrial volume index (LAVI), and tricuspid regurgitation maximum velocity (TRV). Nonetheless, relevant research is limited.

This study aimed to investigate the effects and predictive value of revised DD on post-LT outcomes, based on the revised 2019 CCC criteria.

## Materials and methods

### Study design and participant selection

**Figure 1:**
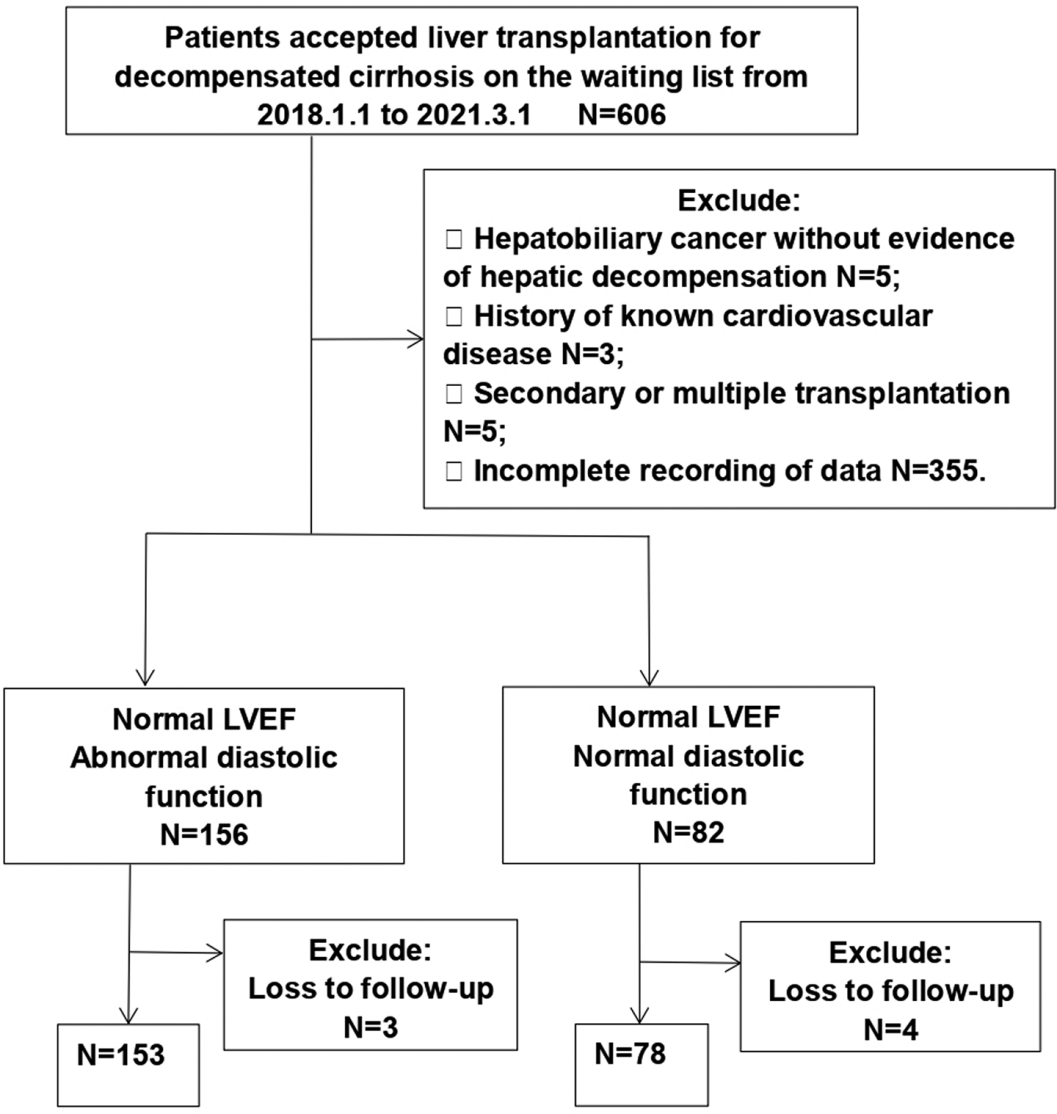
Patient flow diagram.

This retrospective study enrolled patients aged 18–70 years, diagnosed with decompensated cirrhosis (8) at the Affiliated Hospital of Qingdao University, Qingdao, China, between January 2018 and March 2021. Patients were divided into two groups (DD or non-DD group), according to echocardiographic examinations of cardiac diastolic function a week before operation, based on the revised 2019 CCC criteria (9–11). DD was determined if three of the following criteria were met: E/e’ > 15, LAVI > 34 ml/m^2^, e’ < 7 cm/s or TRV > 2.8 m/s. Patients who had known heart disease pre-transplant were excluded. The flow diagram of the study is illustrated in Figure 1. The study was approved by Clinical Trials (NCT04976764) and the Ethics Committee of the Affiliated Hospital of Qing Dao University (QYFYWZLL 26462). All involved persons gave their informed consent (written or verbal, as appropriate) prior to study inclusion.

### Statistical analysis

Enumeration data are presented as frequencies (percentages) and the significance is determined by chi-square test or Fisher's exact test. Intergroup comparisons were performed using a Student's *t*-test or Mann-Whitney *U* test. Multivariate logistic regression analysis models were used to identify independent and predictive factors for poor outcomes. Survival status was assessed using Kaplan–Meier curves and the log-rank test. Significant variables in the univariate analysis were tested in a multivariate analysis using Cox's proportional hazard model, to identify the predictors of survival. The caliper value of propensity score matching (PSM), adjusted for confounders, was set to 0.02. A *P*-value < 0.05 was regarded as statistically significant. SPSS (version 26.0, IBM, New York, USA) was used to analyze the data.

## Results

### Baseline characteristics and serological indexes

Baseline characteristics of the 231 patients were collected (male, 81.8%; mean age 51.5 ± 9.5 years) (Table 1). A total of 153 (66.2%) patients in the DD group were diagnosed with DD with normal left ventricular ejection fraction, according to the 2019 CCC criteria. Before PSM, the patients with DD tended to be older (*P* < 0.01) and were more likely to have hypertension (*P* < 0.01) and diabetes (*P* < 0.05), in comparison to patients without DD. After PSM, there were 97 patients in the DD group and 69 patients in the non-DD group, with no notable differences in sex, age, smoking status, etiology, and basic diseases, including hypertension and diabetes.

**Table 1:** Comparisons of baseline characteristics between patients with and without DD.

	Before PSM			After PSM		
	With DD *N* = 153	Without DD *N* = 78	*P*	With DD *N* = 97	Without DD *N* = 69	*P*
Age			0.004			0.724
<45	26 (17.0%)	24 (30.8%)		23 (23.7%)	17 (24.6%)	
45-	60 (39.2%)	38 (48.7%)		48 (49.5%)	37 (43.6%)	
55-	48 (31.4%)	12 (15.4%)		22 (22.7%)	11 (15.9%)	
65-	19 (12.4%)	4 (5.1%)		4 (4.1%)	4 (5.8%)	
Sex			0.131			0.946
Male	121 (79.1%)	68 (87.2%)		84 (86.6%)	60 (67.0%)	
Female	32 (20.9%)	10 (12.8%)		13 (13.4%)	9 (13.0%)	
BMI			0.074			0.143
<24	58 (37.9%)	38 (48.7%)		37 (38.1%)	35 (50.7%)	
24–27.9	73 (47.7%)	25 (32.1%)		44 (45.4%)	21 (30.4%)	
≥28	22 (14.4%)	15 (19.2%)		16 (16.5%)	13 (18.8%)	
Smoking	51 (33.3%)	33 (42.3%)	0.180	37 (38.1%)	27 (39.1%)	0.898
Alcohol	44 (28.8%)	30 (38.5%)	0.135	31 (32.0%)	25 (36.2%)	0.566
Hypertension	21 (13.7%)	2 (2.6%)	0.007	1 (1.0%)	1 (1.4%)	0.809
Diabetes	38 (24.8%)	9 (11.5%)	0.018	15 (15.5%)	6 (8.7%)	0.196
Anemia			0.073			0.050
No	54 (35.3%)	15 (19.2%)		37 (38.1%)	14 (20.3%)	
Mild	52 (34.0%)	31 (39.7%)		30 (30.9%)	27 (39.1%)	
Moderate	41 (26.8%)	27 (34.6%)		28 (28.9%)	23 (33.3%)	
Severe	6 (3.9%)	5 (6.4%)		2 (2.1%)	5 (7.2%)	
Etiology			0.627			0.854
Alcohol	12 (7.8%)	9 (11.5%)		9 (9.3%)	6 (8.7%)	
Hepatitis B	127 (83.0%)	63 (80.8%)		83 (85.6%)	58 (84.1%)	
Autoimmune	14 (9.2%)	6 (7.7%)		5 (5.2%)	5 (7.2%)	

PSM, propensity score matching; DD, diastolic dysfunction; BMI, body mass index. *P*-value < 0.05 was regarded as statistically significant using Chi-square test or Fisher's exact test.

A comparison of the serological indices and inflammatory markers in Table 2 showed that erythrocyte, hemoglobin, and carbohydrate antigen (CA) 19-9 levels differed significantly between the groups, after PSM (*P* < 0.05). No differences were noted in the systemic immune-inflammation index (SII), platelet-to-lymphocyte ratio (PLR), and neutrophil-to-lymphocyte ratio (NLR) between the groups (*P* > 0.05). Additional results of routine laboratory tests, including complete blood cell counts, liver function, renal function, blood lipids, electrolytes, glucose, myocardial enzymes, coagulation function, and other tumor markers, are described in Supplementary Table S1 of Appendix 1.

**Table 2:** Comparisons of routine laboratory test results and inflammatory markers between patients with and without DD.

	Before PSM			After PSM		
	With DD *N* = 153	Without DD *N* = 78	*P*	With DD *N* = 97	Without DD *N* = 69	*P*
Erythrocytes	3.5 (2.9,4.1)	3.0 (2.6,3.9)	0.007	3.5 (2.9,4.2)	3.0 (2.6,3.9)	0.009
Hemoglobin	107.3 ± 28.0	96.5 ± 23.7	0.004	108.6 ± 28.5	96.5 ± 23.5	0.004
Albumin	31.9 (28.0,36.8)	33.7 (29.0,38.0)	0.040	32.2 (28.1,37.9)	34.3 (29.0,38.0)	0.098
CA199	23.0 (12.5,51.2)	20.0 (7.7,38.5)	0.408	21.8 (9.7,50.7)	16.5 (7.4,34.0)	0.041
Inflammatory markers						
SII	233.7 (143.2,466.3)	239.4 (116.2,582.3)	0.286	240.3 (150.5,476.8)	231.1 (114.9,571.7)	0.681
NLR	2.9 (2.0,5.6)	3.4 (2.2,8.1)	0.256	3.1 (2.1,6.6)	3.4 (1.9,8.6)	0.866
PLR	85.0 (64.9,161.2)	98.3 (69.5,169.0)	0.200	86.0 (62.0,154.1)	96.3 (69.3,162.9)	0.219
PNI	37.1 (30.9,43.6)	38.6 (34.2,45.3)	0.139	37.5 ± 7.9	40.1 ± 7.8	0.187
C-reactive protein	9.2 (2.8,28.8)	13.5 (4.2,27.3)	0.942	8.4 (2.4,33.0)	14.0 (4.0,29.3)	0.969
Procalcitonin	0.7 (0.3,2.4)	0.5 (0.2,1.3)	0.152	0.7 (0.3,2.7)	0.5 (0.2,1.4)	0.112

CA19-9, carbohydrate antigen 19-9; SII, systemic immune-inflammation index; PLR, platelet-to-lymphocyte ratio; NLR, neutrophil-to-lymphocyte ratio; PNI, prognostic nutritional index. *P*-value < 0.05 was regarded as statistically significant using Student's *t*-test or Mann-Whitney *U* test. Please see Appendix 1 Supplementary Table S1 for additional related results of routine laboratory test.

### More echocardiographic abnormalities in patients with DD

There were significant differences in the septal e’, E/e’, and tricuspid regurgitation (TR) maximum velocity between the groups (*P* < 0.01). Moreover, it was found that pulmonary artery systolic pressure, left ventricular pulse wave, and interventricular septum (IVS) were higher in the DD group than in the non-DD group, before and after PSM (*P* < 0.01). The median QT interval was not prolonged (*P* > 0.05). Other echocardiographic characteristics are presented in Table 3.

**Table 3:** Comparisons of echocardiography parameters and QT interval between patients with and without DD.

	Before PSM			After PSM		
	With DD *N* = 153	Without DD *N* = 78	*P*	With DD *N* = 97	Without DD *N* = 69	*P*
LVEF	64.0 (62.0,66.0)	64.0 (62.0,66.0)	0.981	64.0 (62.0,66.0)	64.0 (62.0,66.0)	0.972
e’	6.3 (5.5,6.9)	7.9 (7.3,9.3)	0.000	6.3 (5.5,6.8)	8.0 (7.3,9.5)	0.000
E/e’	11.4 (9.7,12.9)	9.1 (8.2,10.6)	0.000	11.5 (10.0,13.1)	9.3 (8.2,10.0)	0.000
PASP	37.0 (34.8,38.0)	28.0 (25.0,30.0)	0.000	37.0 (34.8,38.0)	28.5 (25.0,30.8)	0.000
TRV	2.8 (2.8,2.9)	2.4 (2.2,2.5)	0.000	2.8 (2.8,2.9)	2.4 (2.2,2.5)	0.000
LAVI	24.7 (20.3,34.3)	22.8 (17.9,29.2)	0.275	24.6 (20.8,33.4)	23.4 (17.7,30.3)	0.350
LVDS	3.1 (2.9,3.2)	3.0 (2.8,3.2)	0.238	3.1 ± 0.3	3.1 ± 0.3	0.224
LVDD	4.8 (4.5,5.0)	4.7 (4.5,5.0)	0.707	4.7 (4.5,4.9)	4.7 (4.6,4.9)	0.309
EDV	108.5 (95.0,121.8)	103.0 (100.3,115.5)	0.051	102.0 (94.0,112.8)	103.0 (100.3,115.5)	0.146
EDT	184.7 ± 53.4	193.4 ± 46.8	0.390	183.0 ± 50.3	190.3 ± 49.6	0.444
LVPW	1.0 (1.0,1.0)	1.0 (1.0,1.0)	0.001	1.0 (1.0,1.0)	1.0 (1.0,1.0)	0.032
IVS	1.0 (1.0,1.2)	1.0 (1.0,1.0)	0.000	1.0 (1.0,1.1)	1.0 (1.0,1.0)	0.003
IVC	15.0 (14.0,16.0)	15.0 (14.5,16.0)	0.209	1.5 (1.5,1.7)	1.5 (1.5,1.7)	0.201
QT	446.3 ± 27.6	441.8 ± 31.7	0.104	403.6 ± 36.6	392.1 ± 29.9	0.149
QTc	444.5 (429.0,463.0)	440.0 (422.0,457.0)	0.085	443.5 (429.0,462.8)	440.0 (420.5,456.5)	0.563
TAPSE	2.2 (2.0,2.5)	2.3 (2.1,2.5)	0.162	2.3 (2.0,2.5)	2.2 (2.1,3.5)	0.117
RVW	0.4 (0.4,0.4)	0.4 (0.4,0.4)	0.978	0.4 (0.4,0.4)	0.4 (0.4,0.4)	0.919
MPA	2.4 (2.2,2.5)	2.3 (2.3,2.3)	0.670	2.4 (2.3,2.5)	2.3 (2.3,2.3)	0.250

LVEF, left ventricular ejection fraction; e’, early diastolic mitral annular tissue velocity; E/e’, early diastolic trans-mitral flow to early diastolic mitral annular tissue velocity; PASP, pulmonary arterial systolic pressure; TRV, tricuspid regurgitation maximum velocity; LAVI, left atrial volume index; LVDS, left ventricle end-systolic internal diameter; LVDD, left ventricle end-diastolic internal diameter; DD, diastolic dysfunction; EDV, end-diastolic volume; EDT, E-wave deceleration time; LVPW, left ventricular posterior wall; IVS, interventricular septum; IVC, inferior vena cava; QTc, QT interval after correction; TAPSE, tricuspid annular plane systolic excursion; RVW, right ventricle wall; MPA, main pulmonary artery; PSM, propensity score matching.

*P*-value < 0.05 was regarded as statistically significant using Student's *t*-test or Mann-Whitney *U* test.

### More serious liver diseases in patients with DD

Patients with DD had an increased Child-Pugh class score before and after PSM (*P* = 0.003 and *P* = 0.045, respectively). No significant differences were observed with respect to cardiac function class, model for end-stage liver disease (MELD) score, physician global assessment score, American Society of Anesthesiology score, bleeding volume, and volume of blood transfusion by PSM (Supplementary Table S2 of Appendix 1). Nonetheless, patients with DD had longer intensive care unit stays than those without DD (*P* < 0.05) (Table 4).

**Table 4:** Comparison of scoring-based estimation and procedure-related data between patients with and without DD.

	Before PSM			After PSM		
	With DD *N* = 153	Without DD *N* = 78	*P*	With DD *N* = 97	Without DD *N* = 69	*P*
Child-Pugh			0.003			0.045
A	22 (14.4%)	25 (32.1%)		14 (14.4%)	21 (30.4%)	
B	90 (58.8%)	31 (39.7%)		51 (52.6%)	29 (42.0%)	
C	41 (26.8%)	22 (28.2%)		32 (33.0%)	19 (27.5%)	
Anhepatic time	53.0 (46.0,60.8)	51.5 (44.3,57.8)	0.220	53.5 (46.0,60.8)	51.0 (42.5,56.5)	0.089
Stay time in ICU	3.0 (3.0,5.0)	3.0 (2.0,4.0)	0.003	3.0 (3.0,5.0)	3.0 (2.0,4.0)	0.001
Cardiovascular adverse events						
Perioperative	24 (15.7%)	5 (6.4%)	0.044	15 (15.5%)	3 (4.3%)	0.023
1-year post-LT	28 (18.3%)	9 (11.5%)	0.185	17 (17.5%)	7 (10.1%)	0.183

DD, diastolic dysfunction; PSM, propensity score matching; ICU, intensive care unit; LT, liver transplantation.

*P*-value < 0.05 was regarded as statistically significant using chi-square test or Mann-Whitney *U* test.

Please see Appendix 1 Supplementary Table S2 for additional scoring-based estimation.

### Poor post-LT outcomes in patients with DD

Enrolled patients were followed up, for at least 1 year. In total, 29 patients (12.6%) developed perioperative cardiovascular adverse events (AEs), 24 of whom had DD. Patients with DD frequently experienced perioperative cardiovascular events (15.7% vs. 6.4%, *P* < 0.05) (Table 4). In a multivariate analysis, anhepatic time (*P* = 0.030), potassium levels (*P* = 0.024), IVS (*P* = 0.018), and bleeding volume (*P* = 0.046) were found to be independent predictors of the incidence of perioperative cardiovascular AEs before PSM. Considering age, sex, etiology, smoking status, and basic diseases, IVS (*P* = 0.008), LAVI (*P* = 0.047), and potassium level (*P* = 0.003) were independently correlated with perioperative cardiovascular AEs after PSM (Table 5).

**Table 5:** Univariable and multivariable logistic regression analysis of perioperative cardiovascular adverse events.

Variable	Univariate analysis		Multivariate analysis	
	OR (95% CI)	*P*	OR (95% CI)	*P*
**(A) Before PSM**				
Etiology		0.011		
Alcohol	Reference			
Hepatitis B	0.667 (0.180–2.473)	0.544		
Immune	3.231 (0.700–14.907)	0.133		
Sex	3.371 (1.452–7.824)	0.005		
BUN	1.182 (1.052–1.329)	0.005		
Anhepatic time	1.031 (1.009–1.054)	0.007	1.033 (1.003–1.063)	0.030
Potassium	1.918 (1.087–3.382)	0.025	2.302 (1.115–4.753)	0.024
Sodium	0.870 (0.801–0.944)	0.001		
Myoglobin	0.998 (0.997–1.000)	0.013		
e’	0.722 (0.520–1.001)	0.051		
E/e’	1.142 (0.976–1.335)	0.097		
IVS	1.802 (1.338–2.427)	0.000	1.594 (1.082–2.347)	0.018
LAVI	1.072 (1.021–1.125)	0.005		
Bleeding volume	1.000 (1.000–1.001)	0.035	1.001 (1.000–1.001)	0.046
Volume of blood transfusion				
Plasma	1.090 (1.033–1.151)	0.002		
Erythrocytes	1.001 (1.000–1.001)	0.021		
**(B) After PSM**				
Sex	4.125 (1.361–12.502)	0.012		
e’	0.558 (0.376–0.829)	0.004		
E/e’	1.289 (1.089–1.526)	0.003		
IVS	2.099 (1.416–3.109)	0.000	2.462 (1.153–5.046)	0.008
LAVI	1.090 (1.033–1.150)	0.002	1.090 (1.001–1.188)	0.047
BUN	1.217 (1.058–1.401)	0.006		
Potassium	2.642 (0.984–7.096)	0.054	14.135 (2.452–81.486)	0.003
Sodium	0.906 (0.819–1.003)	0.057		
Anhepatic time	1.041 (1.012–1.071)	0.005		
Bleeding volume	1.001 (1.000–1.001)	0.063		
Volume of blood transfusion				
Plasma	1.001 (1.000–1.002)	0.002		
Erythrocytes	1.099 (1.025–1.177)	0.008		

BUN, blood urine nitrogen; e’, early diastolic mitral annular tissue velocity; E/e’, early diastolic trans-mitral flow to early diastolic mitral annular tissue velocity; IVS, interventricular septum; LAVI, left atrial volume index; PSM, propensity score matching. *P*-value < 0.05 was regarded as statistically significant using univariable and multivariable logistic regression analysis.

**Figure 2:**
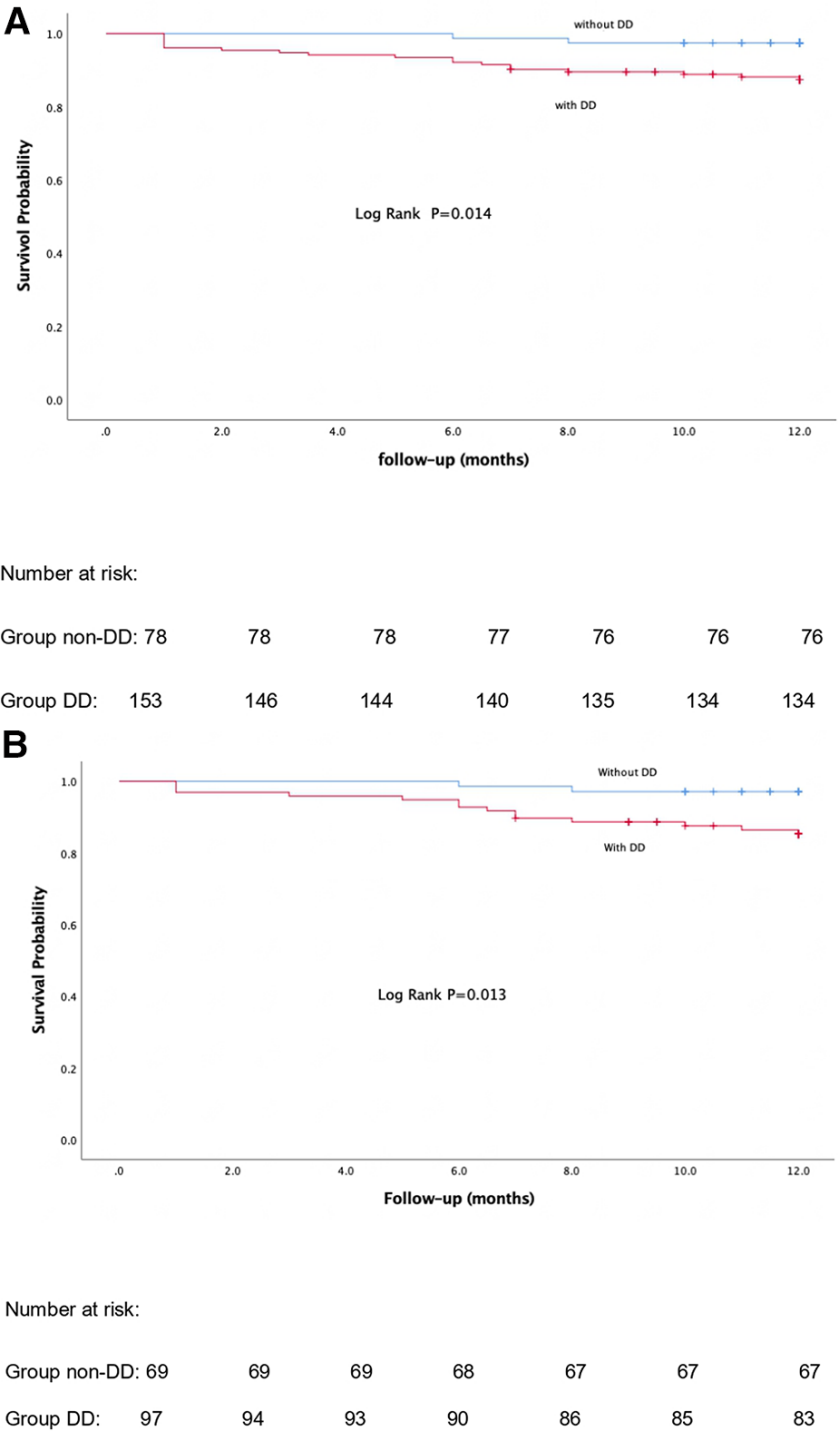
Kaplan-Meier curves of 1-year mortality post-LT before and after propensity score matching analysis. (**A**) Before-PSM. (**B**) After-PSM. *P*-value < 0.05 was regarded as statistically significant.

Twenty-one patients (9.10%) with DD died within the first year of follow-up. The causes were mainly AEs, graft rejection, progression of liver disease and sepsis (Table 6). But we found no statistically significant association between DD and each clinical event. The Kaplan–Meier curves in Figure 2 show that patients with DD had lower survival rates than those without DD. In a multivariable Cox regression analysis, carcinoembryonic antigen, e’, and left ventricle end-systolic internal diameter were correlated with the occurrence of death, within 1-year post-LT. In the model adjusted by PSM, decreased e’, increased NLR and tumor markers were associated with a greater 1-year mortality rate (Table 7).

**Table 6:** Categories of 1-year mortality post-LT.

	Before PSM			After PSM		
	With DD *N* = 153	Without DD *N* = 78	*P*	With DD *N* = 97	Without DD *N* = 69	*P*
Mortality	21 (13.73%)	2 (2.56%)	0.007	14 (14.43%)	2 (2.90%)	0.013
AEs	5 (3.27%)			4 (4.12%)		
Graft rejection	4 (2.61%)			2 (2.06%)		
Progression of liver disease	5 (3.27%)	1 (1.28%)		4 (4.12%)	1 (1.45%)	
Sepsis	6 (3.92%)	1 (1.28%)		4 (4.12%)	1 (1.45%)	
Other	1 (0.65%)					

DD, diastolic dysfunction; AE, adverse events; PSM, propensity score matching.

*P*-value < 0.05 was regarded as statistically significant using Fisher's exact test.

**Table 7:** Univariable and multivariable cox proportional hazard regression analysis of 1-year mortality post-LT.

Variable	Univariate analysis		Multivariate analysis	
	HR (95% CI)	*P*	HR (95% CI)	*P*
**(A) Before PSM**				
Platelet	9.379 (1.048–83.925)	0.002		
Neutrophils	1.006 (1.002–1.009)	0.018		
BUN	1.104 (0.988–1.234)	0.081		
SII	1.002 (1.000–1.004)	0.007		
PLR	1.000 (1.000–1.000)	0.005		
NLR	1.002 (1.001–1.004)	0.017		
CRP	1.001 (1.000–1.002)	0.004		
CEA	1.060 (1.009–1.113)	0.001	1.144 (1.031–1.269)	0.011
e’	0.573 (0.385–0.854)	0.006	0.610 (0.408–0.912)	0.016
LVDS	0.200 (0.045–0.883)	0.034	0.153 (0.028–0.824)	0.029
IVS	1.376 (1.021–1.855)	0.036		
Bleeding volume	1.000 (1.000–1.000)	0.037		
**(B) After PSM**				
Age		0.040		
<45	Reference			
45-	2.800 (0.337–23.256)	0.341		
55-	9.086 (1.118–73.857)	0.039		
65-	10.952 (0.993–120.800)	0.051		
e’	0.545 (0.376–0.788)	0.001	0.570 (0.331–0.981)	0.042
IVS	1.608 (1.179–2.192)	0.003		
Platelet	1.006 (1.003–1.010)	0.000		
SII	1.000 (1.000–1.000)	0.013		
PLR	1.003 (1.001–1.004)	0.005		
NLR	1.055 (0.997–1.118)	0.064	1.023 (1.003–1.043)	0.021
CRP	1.022 (1.008–1.037)	0.003		
AFP	1.000 (1.000–1.000)	0.007	1.000 (1.000–1.000)	0.016
CEA	1.179 (1.076–1.292)	0.000	1.262 (1.009–1.580)	0.042
CA199	1.002 (1.000–1.003)	0.031		
Bleeding volume	1.000 (1.000–1.000)	0.033		

BUN, blood urine nitrogen; SII, systemic immune-inflammation index; PLR, platelet-to-lymphocyte ratio; NLR, neutrophil-to-lymphocyte ratio; CRP, C-reactive protein; CEA, carcinoembryonic antigen; e’, early diastolic mitral annular tissue velocity; LVDS, left ventricle end-systolic internal diameter; IVS, interventricular septum; AFP, alpha-fetoprotein; CA19-9, carbohydrate antigen 19-9; DD, diastolic dysfunction. *P*-value < 0.05 was regarded as statistically significant using univariable and multivariable cox proportional hazard regression analysis.

IVS and e’ were the strongest independent prognostic factors for perioperative cardiovascular AEs and mortality, respectively.

## Discussion

In CCM, an occult onset process upon encountering environmental stress, such as transplantation, may contribute to rapid progression and even significant mortality rates. The primary outcome of this study showed that DD was related to the occurrence of perioperative cardiovascular AEs and mortality, before and after PSM. Among the echocardiographic parameters, IVS, and e’ were the strongest independent prognostic factors for perioperative cardiovascular AEs and mortality, respectively. Upon encountering environmental stress, such as transplantation, impaired contractility fails to adapt to initial changes. Decreased vascular resistance and increased cardiac output increase cardiac preload, resulting in abnormal filling of the ventricle and blood redistribution (12, 13). Histological examination of a heart with CCM revealed edema, cardiomyocyte hypertrophy, nuclear vacuolization, and fibrosis, which occurred in conjunction with enhanced accumulation of collagen. However, we observed that ventricular wall stiffness would partially recover (14–16). Studies have demonstrated that DD increases the risk of cardiovascular AEs post-LT (5, 17). Nonetheless, few studies have reported such correlation based on the 2005 criteria (18).

Furthermore, elevated IVS was the strongest cardiac predictor, increasing the risk of perioperative cardiovascular AEs by 2.4-fold. These results may be attributed to myocardial remodeling, resulting in alterations in the cardiac structure, as onset was earliest in the IVS (19, 20). Decreased e’ aided in confirming which patients were at an increasing risk of poor survival, in both univariate and multivariate regression analysis (6, 21). This may reflect progressive stiffness of the myocardium and deteriorating cardiac function (22). Prior studies have revealed association of E/A, E/e’, and LAVI with poor survival post-LT (17, 23–26); however, it is not well-suited to apply the E/A ratio owing to its load- and age-dependence (27, 28). In comparison, some previous studies have failed to find any relevance (4, 29). The above mentioned studies did not rule out the influence of confounding factors. The present study adopted the 2019 criteria and performed PSM for risk factors, which reduced the bias in the results.

A prolonged QT interval is the main electrophysiological signature of CCM, which is associated with 30-day cardiovascular AEs and mortality, post-LT (30, 31). In the 2019 CCC consensus, QT prolongation was not warranted, because of its limited value in the diagnosis of CCM (32, 33). Potassium is the major predictive factor of perioperative cardiovascular AEs. Ischemia-reperfusion injury post-LT increase the level of extracellular potassium and reduce the concentration gradient between the inside and outside of the cell. Shortening action potential and impaired conductivity was attributed to altered gating of ion channels, predisposing to malignant arrhythmias (34, 35). Because of the limitations of current study population, possibilities of impaired ion channels cannot be completely excluded (11). Pulmonary arterial hypertension has been shown to be involved in liver fibrosis at the gene level and confers higher mortality (36–38). Cardiac dysfunction was first attributed to the direct effect of alcohol; however, it was revealed to be independent of the etiology (39, 40). Cardiac function deteriorated with the progression of cirrhosis, but showed limited progression with stable cirrhosis within 2 years (25, 28, 41). Pro-B-type natriuretic peptides and troponins have also been described as prognostic markers (42, 43). Nevertheless, this was contradicted by the results of our study. In contrast to the Child-Pugh score, the MELD score showed no impact on the presence of DD because of underestimation of the severity of end-stage liver disease on the waiting list for LT (44).

Given the increased risk of infectious complications, upregulation of inflammatory markers and downregulation of cnidarian complements in both the advancement of cirrhosis and development of CCM were associated with poor survival (21, 45–47). Similarly, NLR was considered an effector, along with SII and PLR, in this study. In addition, all enrolled patients had near-normal systolic function at a resting state maintained by the compensatory pathways of hyperdynamic circulatory state and low systemic vascular resistance (48). Additionally, a very low left ventricular ejection fraction is regarded as a contraindication to LT. Prior studies have revealed associations between global longitudinal strain and poor survival (42, 49). Due to the limitations of diagnostic tools, we were unable to further confirm the influence of global longitudinal strain.

Our study has several limitations. First, this was a single-center study, thus lacks representativeness. Moreover, it was limited by its retrospective and observational study design. Therefore, prospective and multicenter validation studies are required.

## Conclusion

Decompensated cirrhosis with DD accelerates perioperative cardiovascular AEs and 1-year post-transplantation mortality rates. Appropriate precedence in decompensated cirrhosis with DD on the waiting list should be considered to ensure timely diagnosis. In PSM analysis, multiple risk factors including IVS, LAVI, e’, potassium, and NLR collectively contributed to perioperative cardiovascular AEs and 1-year mortality, highlighting the need for closer post-LT monitoring and management.

## Data Availability

The original contributions presented in the study are included in the article/Supplementary Material, further inquiries can be directed to the corresponding author.
