# Supplementary material for: The predictive value of revised diastolic dysfunction in outcomes of liver transplantation: A propensity score matching analysis
Source: Front Surg. 2023 Jan 6;9:1072908. doi: 10.3389/fsurg.2022.1072908 (PMC9852983; doi:10.3389/fsurg.2022.1072908)
Supplement: Supplementary file 1 [file Datasheet1.pdf]

CERTIFICATE OF ENGLISH EDITING

This document certifies that the paper listed below has been edited to ensure that the language is clear and free of errors. The logical presentation of ideas and the structure of the paper were also checked during the editing process. The edit was performed by professional editors at Editage, a division of Cactus Communications, in cooperation with Taylor & Francis Group. The intent of the author's message was not altered in any way during the editing process. The quality of the edit has been guaranteed, with the assumption that our suggested changes have been accepted and have not been further altered without the knowledge of our editors.

Title

The Predictive Value of Revised Diastolic Dysfunction in Outcomes of Liver Transplantation: A Propensity Score Matching Analysis

Authors

Shenghua Bi, Yueping Jiang, Wenjun Zhao, Xiaoyan Niu, Xuechun Liu, Xue Jing

Order No.

FRVLS_3_3

EDITINGSERVICES
Supporting Taylor & Francis authors

Signature

Vikas Narang

Vikas Narang,
Chief Operating Officer,
Editage

DNV·GL ISO/IEC 27001

editage

Date of Issue
November 16, 2022
