# Supplementary material for: The predictive value of revised diastolic dysfunction in outcomes of liver transplantation: A propensity score matching analysis
Source: Front Surg. 2023 Jan 6;9:1072908. doi: 10.3389/fsurg.2022.1072908 (PMC9852983; doi:10.3389/fsurg.2022.1072908)
Supplement: Supplementary file 2 [file Datasheet2.pdf]

Table S1. Comparisons of other routine laboratory test results between patients with diastolic dysfunction and controls.

| | Before-PSM | | | After-PSM | | |
|---|---|---|---|---|---|---|
| | **With DD N=153** | **Without DD N=78** | ***P*** | **With DD N=97** | **Without DD N=69** | ***P*** |
| **Complete blood cell count** | | | | | | |
| leukocytes | 3.8 (2.6,5.6) | 4.0 (2.6,5.7) | 0.776 | 3.8 (2.8,6.2) | 3.8 (2.6,5.6) | 0.632 |
| neutrophils | 2.4 (1.4,3.8) | 2.6 (1.6,3.6) | 0.608 | 2.6 (1.5,4.0) | 2.6 (1.5,3.5) | 0.511 |
| lymphocytes | 0.8 (0.5,1.3) | 0.7 (0.5,1.1) | 0.377 | 0.7 (0.5,1.2) | 0.7 (0.5,1.2) | 0.706 |
| platelet | 72.0 (44.0,121.0) | 71.0 (50.5,112.5) | 0.982 | 67.0(42.0,115.5) | 71.0(51.0,109.0) | 0.518 |
| **Liver function** | | | | | | |
| total protein | 61.1±8.8 | 62.0±8.3 | 0.447 | 59.6±8.2 | 61.9±8.0 | 0.070 |
| total bilirubin | 39.2 (22.7,91.8) | 39.8 (20.0,83.5) | 0.848 | 44.2 (25.8,99.9) | 42.4 (22.5,84.8) | 0.389 |
| direct bilirubin | 17.5 (9.0,48.4) | 19.1 (9.1,42.7) | 0.986 | 20.9 (11.1,50.4) | 19.1 (10.2,43.3) | 0.516 |
| total bile acid | 36.8 (14.3,100.0) | 32.5 (9.1,87.6) | 0.263 | 39.7 (14.8,100.0) | 39.8 (9.4,91.3) | 0.463 |
| ALT | 32.0 (19.5,62.5) | 32.0 (19.0,61.5) | 0.997 | 36.0 (21.0,73.0) | 32.0 (19.0,69.5) | 0.739 |
| AST | 42.0 (27.0,72.0) | 37.5 (23.0,88.3) | 0.710 | 47.0 (28.5,72.5) | 40.0 (25.5,128.5) | 0.946 |
| ALT/AST | 0.8 (0.6,1.0) | 0.8 (0.6,1.0) | 0.727 | 0.8 (0.6,1.1) | 0.8 (0.5,1.0) | 0.245 |
| alkaline phosphatase | 86.0 (60.0,133.0) | 86.0 (58.0,127.0) | 0.581 | 83.0 (59.0,138.0) | 89.0 (58.0,128.0) | 0.870 |
| lactic dehydrogenase | 196.0(168.5,257.0) | 188.5 (152.8,267.5) | 0.413 | 196.0 (169.0,228.0) | 202.0(158.0,278.0) | 0.991 |
| α-L-Fucosidase | 30.0 (20.0,37.5) | 26.0 (19.8,39.3) | 0.603 | 29.0 (20.0,37.0) | 26.0 (21.0,40.0) | 0.984 |
| blood ammonia | 39.5 (25.0,61.3) | 35.0 (22.0,62.0) | 0.453 | 40.0 (24.0,60.0) | 35.0 (22.0,55.0) | 0.384 |
| **Renal function** | | | | | | |
| Cr | 74.0 (63.0,88.0) | 78.0 (67.0,88.1) | 0.748 | 72.0 (63.0,86.0) | 77.0 (63.5,88.0) | 0.325 |
| BUN | 4.8 (3.9,6.4) | 4.8 (3.8,6.5) | 0.578 | 4.6 (3.8,6.2) | 4.8 (3.9,6.2) | 0.669 |
| BUN/Cr | 16.5 (13.2,21.7) | 15.5 (12.8,22.5) | 0.590 | 17.1 (13.1,21.8) | 17.1 (12.9,23.2) | 0.984 |
| uric acid | 270.0 (158.0,347.0) | 273.0 (212.5,338.8) | 0.253 | 253.8±119.2 | 280.6±106.8 | 0.137 |
| **Blood lipid** | | | | | | |
| triglyceride | 0.8 (0.6,1.3) | 0.8 (0.5,1.2) | 0.619 | 0.8 (0.5,1.3) | 0.8 (0.5,1.4) | 0.889 |
| total cholesterol | 2.6 (1.7,3.8) | 2.4 (1.7,3.3) | 0.178 | 2.5 (1.6,3.8) | 2.3 (1.7,3.2) | 0.562 |
| VLDL cholesterol | 1.4 (0.8,2.1) | 1.2 (0.7,1.8) | 0.431 | 1.2 (0.8,2.0) | 1.2 (0.7,1.7) | 0.419 |
| HDL cholesterol | 0.7 (0.5,1.1) | 0.7 (0.5,1.0) | 0.125 | 0.7 (0.5,1.2) | 0.7 (0.5,1.0) | 0.585 |
| apolipoprotein A1 | 0.7 (0.5,1.0) | 0.7 (0.5,0.8) | 0.182 | 0.7 (0.5,1.0) | 0.7 (0.5,1.0) | 0.232 |
| **Glucose** | 5.5 (4.6,8.5) | 5.9 (4.5,8.4) | 0.935 | 5.4 (4.6,8.1) | 6.0 (4.6,7.9) | 0.497 |
| **Electrolytic** | | | | | | |
| potassium | 3.8 (3.5,4.2) | 3.9 (3.6,4.2) | 0.447 | 3.8 (3.6,4.2) | 3.9 (3.6,4.3) | 0.944 |
| sodium | 141.0 (138.0,144.0) | 141.5 (139.0,144.0) | 0.795 | 140.5±4.7 | 141.2±4.9 | 0.973 |
| chlorine | 106.9(103.0,109.0) | 107.0 (103.0,109.1) | 0.868 | 106.0±4.7 | 105.7±4.9 | 0.630 |

| | | | | | | |
|---|---|---|---|---|---|---|
| calcium | 2.2 (2.0,2.4) | 2.3 (2.1,2.4) | 0.887 | 2.2 (2.0,2.4) | 2.3 (2.2,2.4) | 0.380 |
| **Myocardial enzyme** | | | | | | |
| Myoglobin | 158.0 (35.5,704.0) | 278.5 (27.0,724.5) | 0.751 | 256.0 (36.4,751.7) | 226.2 (27.0,699.4) | 0.228 |
| pro-BNP | 148.0 (63.0,323.3) | 149.0 (52.4,286.2) | 0.650 | 141.0 (51.6,332.5) | 146.0 (45.9,263.4) | 0.470 |
| troponin | 0.02 (0.00,0.09) | 0.03 (0.01,0.10) | 0.502 | 0.02 (0.00,0.09) | 0.02 (0.00,0.07) | 0.613 |
| **Coagulation function** | | | | | | |
| PT | 15.6 (13.1,19.9) | 16.2 (13.4,19.2) | 0.892 | 16.8 (13.5,20.6) | 16.0 (13.4,19.2) | 0.252 |
| INR | 1.3 (1.1,1.7) | 1.3 (1.1,1.6) | 0.994 | 1.4 (1.1,1.9) | 1.3 (1.1,1.6) | 0.238 |
| D-Dimer | 690.0 (320.0,1600.0) | 740.0 (315.0,1692.5) | 0.792 | 740.0 (310.0,1740.0) | 740.0 (310.0,1695.0) | 0.946 |
| **Tumor markers** | | | | | | |
| AFP | 10.0 (2.5,72.5) | 5.0 (2.0,19.9) | 0.081 | 10.9 (2.5,494.9) | 5.1 (2.2,15.3) | 0.162 |
| CEA | 2.6 (1.6,3.8) | 2.4 (1.3,4.0) | 0.226 | 2.4 (1.5,3.7) | 2.4 (1.4,3.8) | 0.413 |
| CA125 | 54.3(18.5,165.7) | 54.5 (14.9,122.1) | 0.408 | 43.1(15.8,115.8) | 51.9 (14.3,120.1) | 0.973 |

ALT, alanine aminotransferase. AST, Aspartate aminotransferase. Cr, creatinine. BUN, blood urine nitrogen. PT, Prothrombin time. INR, international normalized ratio. AFP, Alpha-fetoprotein. CEA, carcinoembryonic antigen. CA125, Carbohydrate Antigen 125.

P-value < 0.05 was regarded as statistically significant using Student's t-test or Mann-Whitney U test.

Table S2. Comparisons of other scoring-based estimation between patients with diastolic dysfunction and controls.

| | Before-PSM | | | After-PSM | | |
|---|---|---|---|---|---|---|
| | **With DD N=153** | **Without DD N=78** | ***P*** | **With DD N=97** | **Without DD N=69** | ***P*** |
| MELD | | | 0.270 | | | 0.453 |
| low risk | 133 (86.9%) | 69 (88.5%) | | 82 (84.5%) | 62 (89.9%) | |
| middle risk | 5 (3.3%) | 5 (6.4%) | | 4 (4.1%) | 3 (4.3%) | |
| high risk | 15 (9.8%) | 4 (5.1%) | | 11 (11.3%) | 4 (5.8%) | |
| PGA index | 4.0 (3.0,5.0) | 4.0 (3.0,5.0) | 0.483 | 4.0 (3.0,5.0) | 4.0 (3.0,5.0) | 0.351 |
| ASA class | | | 0.903 | | | 0.853 |
| I+II | 4 (2.6%) | 1 (1.3%) | | 2 (2.1%) | 1 (1.4%) | |
| III | 96 (62.7%) | 48 (61.5%) | | 66 (68.0%) | 43 (62.3%) | |
| IV | 44 (28.8%) | 24 (30.8%) | | 23 (23.7%) | 20 (29.0%) | |
| V | 9 (5.9%) | 5 (6.4%) | | 6 (6.2%) | 5 (7.2%) | |
| Cardiac function | | | **0.006** | | | 0.078 |
| I+II | 125 (82.4%) | 74 (94.9%) | | 83 (85.6%) | 65 (94.2%) | |
| III+IV | 28 (17.6%) | 4 (5.1%) | | 14 (14.4%) | 4 (5.8%) | |
| Bleeding volume | 800.0 (600.0,1000.0) | 800.0 (600.0,1450.0) | 0.487 | 800.0 (600.0,1150.0) | 800.0 (600.0,1400.0) | 0.773 |
| Volume of blood transfusion | | | | | | |
| plasma | 970.0 (725.0,1047.5) | 970.0 (775.0,1000.0) | 0.375 | 970.0 (777.5,1047.5) | 970.0 (770.0,1000.0) | 0.647 |
| erythrocytes | 7.5 (2.1,11.5) | 8.0 (4.0,11.9) | 0.882 | 7.5 (2.0,11.9) | 8.0 (4.0,11.8) | 0.293 |

MELD, Model for end-stage liver disease. PGA, physician global assessment. ASA, American Society of Anesthesiology.

*P*-value < 0.05 was regarded as statistically significant using Chi-square test or Fisher's exact test, and Mann-Whitney U test.
